# Physical optimization of cell proliferation and differentiation using spinner flask and microcarriers

**DOI:** 10.1186/s13568-022-01397-8

**Published:** 2022-05-31

**Authors:** Feng Yang, Shouwei Wang, Yingying Li, Shilei Li, Wenting Liu, Yushuang Li, Haijuan Hu

**Affiliations:** 1 China Meat Research Center, Beijing, 100068 China; 2 Beijing Institute of Food Science, Beijing, 100068 China

**Keywords:** Cell proliferation, Cell-based meat, Myogenesis, Spontaneous contraction, Microcarriers, Spinner flask, Optimized culture system

## Abstract

**Abstract:**

The traditional breeding industry has been increasingly saturated and caused environmental pollution, disease transmission, excessive resource use, and methane emission; however, it still cannot meet the needs of the growing population. To explore other alternatives, researchers focused on cell agriculture and cell-based meat, especially large-scale cell culture. As a prerequisite for production, large-scale culture technology has become an important bottleneck restricting cell-based meat industrialization. In this study, the single-factor variable method was adopted to examine the influence of Cytodex1 microcarrier pretreatment, spinner flask reaction vessel, cell culture medium, serum and cell incubation, and other influencing factors on large-scale cell cultures to identify the optimization parameters suitable for 3D culture environment. Collagen and 3D culture were also prospectively explored to promote myogenesis and cultivate tissue-like muscle fibers that contract spontaneously. This research lays a theoretical foundation and an exploratory practice for large-scale cell cultures and provides a study reference for the microenvironment of myoblast culture in vitro, a feasible direction for the cell therapy of muscular dystrophy, and prerequisites for the industrialized manufacturing of cell-based meat.

**Graphical Abstract:**

**Figure:**
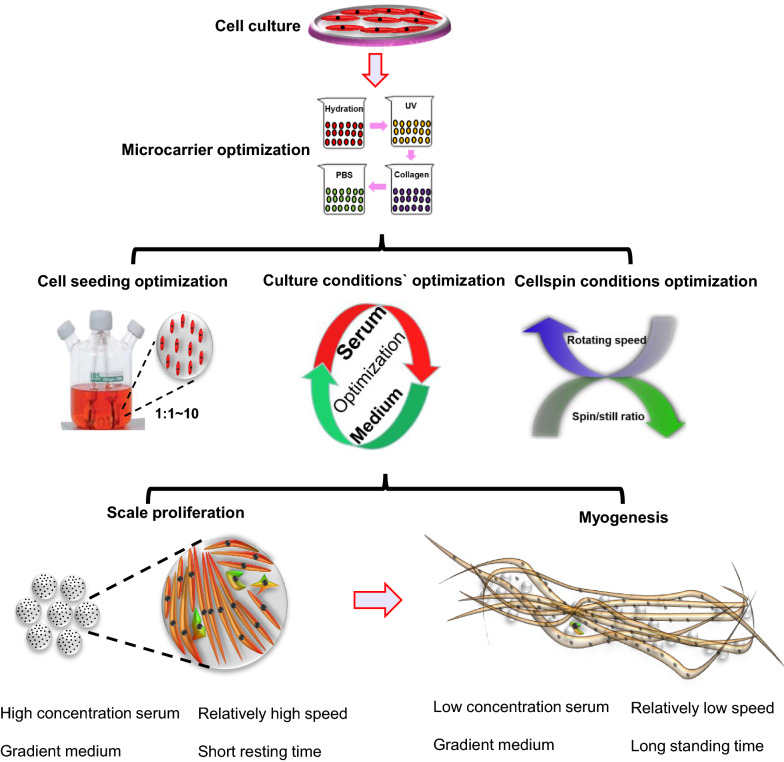
Graphical summary: Research on large-scale myoblast culture using spinner flasks and microcarriers. For cell culture, the microcarriers were pretreated with UV and collagen. Cell seeding condition, spinner flask speed, resting time, and spinner flask culture microenvironment were then optimized. Finally, two culture systems were prepared: a culture system based on large-scale cell expansion and a culture system for myogenesis promotion and differentiation

**Supplementary Information:**

The online version contains supplementary material available at 10.1186/s13568-022-01397-8.

## Introduction

Cell cultures separate cells from animals and cultivate them in vitro to achieve growth and self-reproduction (Gospodarowicz et al. 1980; Huang et al. 2010; Merten, 2006). Cell cultures in vitro have been widely used (Li et al. 2012; Muller et al. 2000; Zhang et al. 1991) and applied to many fields, including vaccines (Perdue et al. 2011), hormones (Pattillo et al. 1968), nucleic acid (Geall et al. 2013), antibodies (Birch and Racher, 2006), and enzyme production (Krause et al. 2010) depending on the animal cell structure, function, and differentiation. The above biological factors and preparations require a large-scale production (Chu and Robinson, 2001); however, the scale of industrial development is limited by the relatively large surface space required for cell adherence culture. Whether cells can efficiently attach to the culture surface is determined by their characteristics and the effective contact probability and biocompatibility level between them and the carrier surface (Hynes, 1999; Maheshwari et al. 2000; Walker et al. 2005; Yu et al. 2020). Traditional petri dishes are suitable for basic scientific research. For large-scale cell culture, specific surface area utilization has become a bottleneck problem. Therefore, researchers focused on cell growth microcarriers that can be used for the 3D culture of cells in a planar growth mode (Lee et al. 2008) to improve the efficiency of cell culture, reduce the culture space, and increase the utilization rate of specific surface area. However, only a few reports are available on dedicated microcarriers for large-scale myoblast culture. Moreover, the existing microcarriers have been used for all adherent cells, and the relevant optimization conditions have not been established.

In microcarrier-based cell cultures, cells attach to the surface of microcarriers to increase their number and volume and reduce the specific gravity of microcarriers to create a suspension culture (Derakhti et al. 2019). Microcarriers have been extensively studied, and their materials mainly include chitosan (Huang et al. 2018), hydrogel (Dias et al. 2017), alginate (Chui et al. 2019), collagen (CG) and hyaluronic acid (Lai, 2014; Overstreet et al. 2003) and polyethylene. The availability of microcarriers is mainly reflected in their mechanical properties (Tavassoli et al. 2018), cell compatibility (Thonhoff et al. 2008), sedimentation coefficient (Pollack et al. 2000), degradation kinetics, and stimulation of physical and chemical properties in the body. Microcarriers are categorized by cell diameter (50–100 um for embryonic stem cells, 100–300 um for mesenchymal stem cells, and 200–600 um for multi-cell mixture) and are used depending on the cell type (Turner and Flynn, 2012). In large-scale cell cultures, the culture efficiency of microcarriers varies for different research purposes, cell types, and culture environments (Chen et al. 2015). At present, only a few microcarriers used in cell-based meat can be directly applied in myoblast proliferation and differentiation.

Optimizing the conditions of microcarrier-based cell cultures is important for its large-scale application (Chen et al. 2015; Derakhti et al. 2019; Forestell et al. 1992). One method is to construct a cell micro-ecosystem using microcarriers, biological reaction vessels, cells, and culture media. The spinner flask reaction vessel is widely used in cell cultures because it can provide a homogeneous culture environment (Song et al. 2007). During culture, cells are also affected by extracellular factors, such as culture conditions, spinner flask speed, resting time, and type of microcarriers (Melke et al. 2018). To date, only a few reports are available on the optimization of large-scale culture and myogenesis of myoblasts for cell-based meat production. In the current work, Cytodex1 microcarriers were applied to explore the influence of several factors such as microcarrier pretreatment, roller flask reaction vessel, cell culture medium, serum, and cell incubation on the large-scale expansion of cells and determine suitable parameters for the 3D optimization of cell culture by using single-factor variable method. CG and 3D cultures were also prospectively explored to promote myogenesis and cultivated tissue-like muscle fibers that contract spontaneously. This research lays a theoretical foundation and an exploratory practice for large-scale cell cultures and provides a study reference for the microenvironment of myoblast culture in vitro, a feasible direction for the cell therapy of muscular dystrophy, and prerequisites for the industrialized manufacturing of cell-based meat.

## Methods

Cells were obtained from 25-day-old embryos of Bailaihang broilers. All experimental procedures were approved by the Experimental Animal Management Committee of Beijing University of Agriculture. Animal experiments were conducted in compliance with the Beijing University of Agriculture Animal Welfare and Ethical Review with experimental facility certification number SYXK 2015-0004. This study was reported in accordance with ARRIVE guidelines.

For cell culture, muscle tissues were cut into small pieces and digested with 0.25% trypsin for 30 min. Mechanical dissociation was performed every 5 min in the middle. A threefold volume of culture medium or onefold volume of fetal bovine serum was added to terminate the digestion. The digestion solution was then collected and centrifuged at 1500 r/min for 5 min. The supernatant was removed, and the cells were resuspended in a new medium and transferred to a culture plate or flask for culture. The medium was changed for the first time in 24 h, and cell growth status was observed. The cells were passed after reaching 90% confluence.

Optimization of microcarrier pretreatment conditions: the microcarriers were swelled with PBS (Invitrogen, USA). After 24 h, the microcarriers were exposed to UV radiation for 30 min and then transferred to 20% CG in PBS for 1 h. Pretreatment control: 25 ml (10^6^ pcs) of swelled and hydrated microcarriers (Cytodex 1, China) were mixed with cells in a spinner flask at a speed of 25 rpm/min. After 2 h, 100 ul of medium was obtained from the culture system, and the number of cells attached to the microcarriers after tenfold dilution was counted and averaged. The number of cells on the microcarriers was used as the number of inoculated cells to adhere to the wall. After 24 h, 100 ul of medium was drawn from the culture system and diluted 10 times. The attachment efficiency of cells in the microcarriers was then examined.

Optimization of serum conditions: the myoblasts were placed in a normal culture dish and digested with 0.25% trypsin for 4 min. A threefold volume of normal medium was added to terminate the digestion. The cells were collected in a 15 ml centrifuge tube and centrifuged at 1000 rpm for 5 min, and the supernatant was removed. Media with serum concentrations of 10%, 20%, 30%, 40% and 50% were then added. The cells were resuspended and transferred to a spinner flask at a speed of 25 rpm/min. After each rotation for 5 min, the solution was allowed to stand for 40 min. After six cycles, 100 ul of the microcarrier mixture was obtained and diluted 10 times. The status of the cells attached to the microcarriers and the number of attached cells were observed under a microscope. For cell counting, the total number of attached cells and the number of microcarriers were counted under a microscope, and the quotient and average number of cells attached to each microcarrier were calculated. A serum-free medium was then to the culture system every 24 h to dilute the serum concentration until a final concentration of 10%. For the medium with 10% serum concentration, the medium was added or replaced with a 10% serum concentration. For the control group, the serum-free culture condition was set as the control to determine the influence of serum on the cells, and the 10% serum concentration medium was set as the control to analyze the effect of different serum concentrations on cell attachment efficiency. Other conditions were the same as those in the serum optimization group.

Spinner flask resting time optimization: spinner flask rotation allows the cells to fully mix with the microcarrier, and standing allows the cells to attach to the surface of the microcarrier. In this study, the myoblasts in the culture dish were first digested with 0.25% trypsin for 4 min, and then a threefold volume of medium were added to terminate the digestion. The cells were transferred to a 15 ml centrifuge tube, centrifuged at 1000 rpm for 5 min, resuspended, and added with the microcarriers for 15 min. After the microcarrier sunk into the bottom of the centrifuge tube, the supernatant was aspirated, 10% fetal bovine serum medium was added, and the solution was transferred to the spinner flask. The rotation time was set to 5 min, and the resting time was set to 10, 20, 40, 60 and 80 min. Other conditions remained constant. In brief, 100 ul of culture medium was collected every 2 h, and the average numbers of cells on each microcarrier and clumps produced by cell adhesion were observed counted on each 4 × field of view of a microscope.

Medium optimization system: a medium with highly concentrated serum is conducive to cell attachment and proliferation but increases the culture cost. This research first optimized cell attachment and proliferation by increasing the serum concentration. The medium system was optimized after the cells had completely attached to the microcarriers. The specific optimization procedure was as follows. After 2 h of culture in highly concentrated serum, the serum-free medium was added for the first time at an amount of 25% of the current culture system volume. After 12 h of culture, half of the serum-free medium of the current culture system was then added. After 24 h of culture, another serum-free medium was added until the final serum concentration of the culture system reached 10%. For the culture system with an initial serum concentration of 10%, a corresponding volume of medium with a serum concentration of 10% was added. For any medium with highly concentrated serum, the addition was stopped when the serum concentration in the culture system reached 10%, and the serum-free medium was replaced with a medium with 10% serum concentration.

The number of inoculated cells: for the culture of microcarriers and spinner flasks, the number of inoculated cells substantially affects the efficiency of cell culture. A small number of inoculated cells either prolongs or inhibit cell proliferation, and an excessive number reduces the efficiency of microcarriers. In this study, cells were inoculated with microcarriers at different ratios to explore the optimal number of inoculated cells in microcarrier spinner flask culture. First, the microcarriers were incubated with PBS, resuspended, and counted. A total of 106 microcarriers were added to the spinner flask system. The cell factory (large petri dish) was added with 10 ml of 0.25% trypsin to digest the cells for 3 min and then with 30 ml of medium to terminate the digestion. The cells were transferred to a 50 ml centrifuge tube and centrifuged at 1000 rpm for 5 min. The supernatant was removed, and the culture medium was added to resuspend the cells and count them with a cell counter. Afterward, 1*10^6^, 2*10^6^, 4*10^6^, 8*10^6^ and 1*10^7^ cells were added to the spinner flask culture system. The rotating speed of the spinner flask was set to 25 rpm/min, rotated for 5 min, and then allowed to stand for 40 min. In brief, 1 ml of the microcarrier and cell mixture system was extracted every 24 h and centrifuged at 1000 rpm for 5 min. The supernatant was removed, and 1 ml of 0.25% trypsin was added to digest the cells for 4 min, followed by 3 ml of the medium to terminate the digestion. The cell and microcarrier mixture was resuspended and separated through differential centrifugation (centrifuge at 500 rpm for 30 s, transfer the supernatant to a new 15 ml centrifuge tube at 1000 rpm for 5 min, aspirate the supernatant, and add 1 ml of culture medium to resuspend the cells). The cells were counted with a cell counting instrument, and the number of cell proliferation was continuously counted until the 10th day to draw a cell growth curve.

For cell staining: the microcarriers and cells were washed with PBS three times. PBS was removed, and 4% paraformaldehyde (Solarbio, China) was added for fixation for 10 min. The samples were washed twice with PBS, added with 0.1% Triton-100 (Sigma, USA) to break the membrane for 5 min, washed twice with PBS, added with 3 ml of phalloidin staining working solution (Absin, China), stained for 30 min, washed twice with PBS (10 min), stained with DAPI working solution (Sigma, USA) for 8 min, washed twice with PBS (5 min), and observed under a fluorescence microscope. For cell counting: the cells on the microcarriers were first digested with trypsin (Gibco, USA), collected, and then counted using a cell counter. The number of cells on each microcarrier was counted and recorded by taking pictures with a microscope to calculate cell attachment efficiency.

For data analysis: original data were obtained through microscopic counting and subjected to statistical analysis and graphing with R.

## Results

### Microcarrier pretreatment improves cell adhesion efficiency

**Fig. 1:**
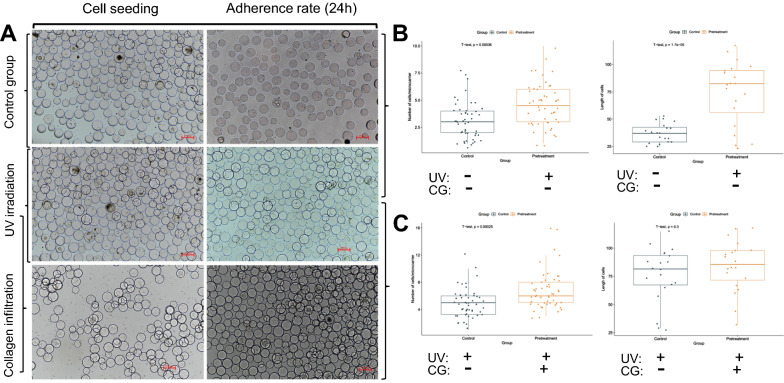
Effect of cell pretreatment on cell attachment and proliferation. **A** Control, UV irradiation, and collagen infiltration three groups of cell inoculation status and 24 h attachment efficiency results. **B** Comparison results of cell attachment efficiency between the control group and the UV treatment group. **C** UV and/or CG treatment compares the results of cell attachment efficiency with the control group

The infiltrated microcarriers were treated first with UV (Inam Ul et al. 2014) and then CG to explore the effects of microcarrier pretreatment on cells (O'Brien et al. 2005). The results showed (Fig. 1) that in the control group (inoculated cells after PBS infiltration), only a few inoculated cells were attached to the microcarriers (Fig. 1, A top), and most were in a free state in the culture medium. The number of cells in the UV group (Fig. 1A) was significantly higher than that in the control group; however, cell dissociation occurred, and the state of free cells was poor. The number of cells attached to the microcarriers in the UV+ CG group (Fig. 1, under A) was higher than that in the UV group. Although some cells were in a free state, the cell status was relatively good from the morphological point of view, and further attachment was still possible. This finding indicated that UV improves microcarrier state and cell adhesion but damages the cells. Meanwhile, CG promotes cell adhesion, reduces UV damage to cells, and increases cell activity thereby cell attachment. After 24 h of inoculation, the cells attached to the microcarriers were counted microscopically (the microcarriers without cell attachment were not involved in the counting). The results showed that the number of cells attached to the UV-treated microcarriers was significantly higher than that of the control group (Fig. 1B, left). At same time, differences in cell length were observed (Fig. 1B, right). The cell length in the UV+ CG group was significantly higher than that in the UV group (Fig. 1 C, left), but the difference was not significant (Fig. 1 C, right). In summary, UV and CG treatments can increase cell attachment to the microcarriers and are conducive to cell expansion and proliferation. Although cell viability cannot be perfectly interpreted, cell stretch state and length can reflect the attachment, stretching, and proliferation of cells attached to the microcarriers. Therefore, microcarriers can be treated with UV and CG prior to cell inoculation to improve cell adhesion efficiency and normal cell expansion for efficient cell proliferation. In addition, the number of clumps produced in the later stage of the cells increased with CG concentration, implying that a certain CG concentration can promote myogenesis.

### Serum optimization can promote cell adhesion and cell status

**Fig. 2:**
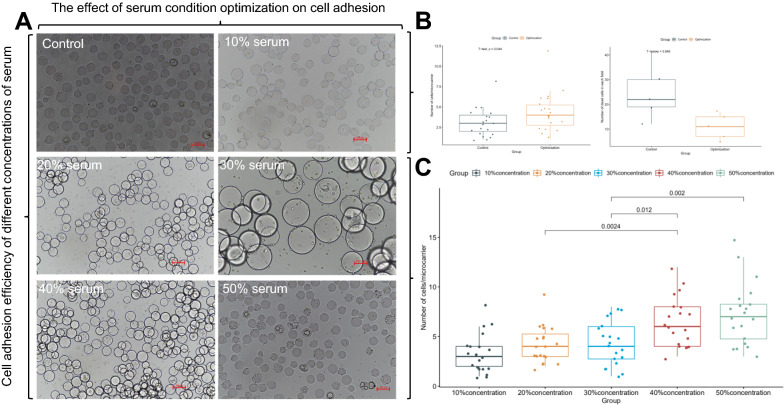
Effect of serum condition optimization on cell adhesion. **A** Effect of serum-free, 10%, 20%, 30%, 40%, and 50% serum concentrations on the efficiency of cell attachment. **B** Comparison of the number of attached and dead cells between the optimized serum and control groups. **C** Difference in the number of cell attachments in media with different serum concentrations

In vitro culture, the serum must contain a large number of growth factors to maintain cell viability and promote cell growth. High serum concentrations can sustain the good state of cells and improve their proliferation. Here, cell culture optimization was performed by gradually adding the medium to serially dilute the highly concentrated serum (the initial highly concentrated serum was gradually added with the medium to achieve 10% dilution during culture). The effect of different serum concentrations on the cells was then analyzed to explore a suitable culture method (Fig. 2). First, the role of serum in cell cultures was verified (Fig. 2A). The results showed that in the control group, the number of adherent cells was significantly lower than that in the 10% serum group (Fig. 2, B left), and the number of dead cells (irregular damaged cells visible in each field of view) was significantly higher than that in 10% serum group. This finding revealed the important role of serum in cell cultures. The effects of different initial serum concentrations on the cells were then examined. Cell morphology results (Fig. 2, A 10–50%) showed that when the initial serum concentration increased, the number of cell attachments also increased significantly, and the cell condition was significantly improved. The number of adherent cells on each microcarrier was counted (Fig. 2C). With increasing serum concentration, the average number of adherent cells on each microcarrier also increased significantly. In particular, the 40% initial serum concentration group had a significantly higher average number of adherent cells than the 20% group, and the 50% group had a higher number than the 30% group. On the basis of the difference in the number of attached cells and the cost of culture, 40% initial serum concentration is the suitable serum optimization plan. Cell morphology results (Fig. 2, A 40–50% serum) also showed that the cells in 50% initial serum concentration stretched, attached, and proliferated earlier than those in 40%. No difference in cell morphology was found between the 40% and lower groups. On the basis of the above results, the optimized 50% initial serum concentration is suitable for subsequent experiments.

### High rotation speed and short resting time of spinner flask promote cell attachment and proliferation

**Fig. 3:**
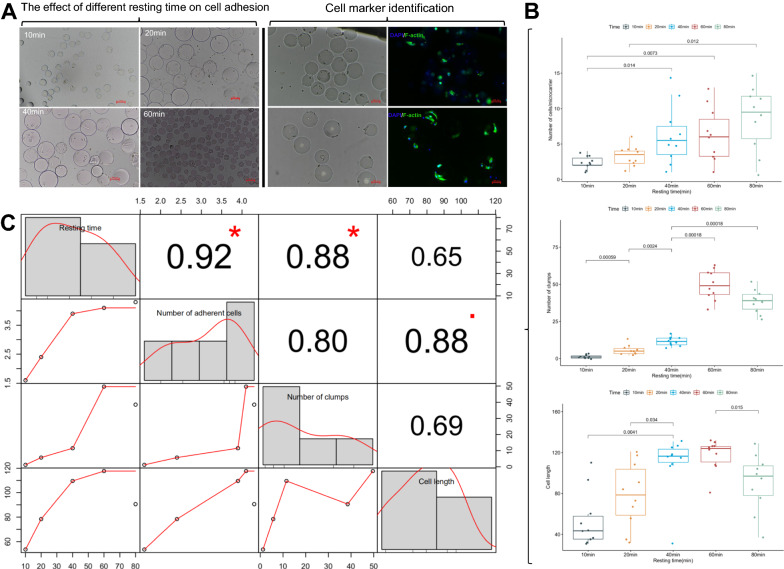
Effect of different resting times on cell adhesion. **A** Cell attachment and morphological results. **B** Comparative analysis of the number of attached cells and produced clumps and the difference in cell length in the cell culture microenvironment at different resting times. **C** Multi-factor correlation analysis for resting time, number of attached cells, number of clumps, and length of cells

In spinner flask cell culture, the microcarriers must reach a state of weightlessness without excessive shearing force. Hence, the culture conditions of different cell types must be adjusted accordingly. In this study, different rotation speeds and spin–stop ratios (resting time) were set to explore the changing law of myoblast adherence and proliferation and reveal the method suitable for the large-scale proliferation of myoblasts. First, the minimum rotation speed to suspend the microcarriers through different rotation speed experiment groups was examined. The results showed that when the rotor of the spinner flask reached 30 rpm/min, the microcarriers loaded with myoblasts can achieve weightlessness in suspension, and the shear force of the medium was relatively small and therefore did not affect the normal state of cells. Resting time is rotation time: also an important parameter that affects cell attachment and proliferation. The excessive resting time will cause clumps, and insufficient resting time will cause poor cell attachment. After different resting time gradients were applied (10, 20, 40, 60 and 80 min), the number of attached cells, number of clumps, and length of the cells were analyzed. The results showed (Fig. 3) significant differences in the number of cell attachments at different resting times (Fig. 3, A left). When the resting time increased from 10 to 60 min, the number of cell attachments also increased significantly. Cell morphological identification by protein markers (Fig. 3, A, right) revealed that the morphology of cells resting for 60 min was significantly better than that of the cells resting for 20 min. Cell length measurement and microscopic counting of the cells and clumps of the microcarriers with different resting times were conducted to further analyze the differences in the cell attachment of microcarriers with different resting times (Fig. 3B). The results showed (Fig. 3, B top) that the microcarriers rested for 40 and 60 min had a significantly higher number of attached cells than those rested for 10 min. In particular, the microcarriers that rested for 80 min had a significantly higher number of attached cells than those that rested for 20, 40 and 60 min. After the microcarriers were suspended, at least 40 min of inactivity improved the efficiency of cell attachment. The number of clumps produced at different resting times was also counted under the quadruple lens field (Fig. 3B). The number of clumps in the culture medium increased with resting time. In particular, the number of lumps in the spinner flask placed for 10, 20, 40 and 60 min gradually increased, and the difference was extremely significant. The clumps in the 80 min group were had a significantly lower number but greater volume than those in the 60 min group. Owing to the long resting time, the cells interacted and consequently promoted myogenesis in the microenvironment, thereby producing muscle fibers. The muscle fibers contacted, adhered, interacted, and eventually formed large clumps. The number of clumps in the 60 and 80 min groups was significantly higher than that in the 10, 20, and 40 min groups. In general, the number of produced clumps was less when the resting time was ≤ 40 min. Cell morphology and length were then analyzed (measure the longest position of the cell). The results showed (Fig. 3, bottom B) that with the increase in resting time, the cells stretched and became elongated. In particular, the cells were significantly longer in the 40 min group than in the 10 and 20 min groups and in the 60 min group than in the 80 min group. In summary, with the increase in resting time, the cell state is well maintained, and attachment and stretching are promoted. When the resting time exceeded 60 min, the number of attached cells increased. The single cells occupied small specific surface areas of the microcarriers, leading to contact inhibition. As a result, the cells cannot be fully stretched. Finally, correlation analysis was conducted for the number of attached cells, number of clumps, and length of cells in different resting times. The results showed (Fig. 3C) that resting time had a significant positive correlation to the number of attached cells (r = 0.92) and clumps (r = 0.88). Cell length and resting time also showed a positive correlation in stages (r = 0.65). A significant negative correlation was also found between the length and number of attached cells (r = 0.88). On the basis of the above results, 40 min is the best resting time for microcarrier-based myoblast culture.

### Low rotation speed and long standing time of the rotating bottle promote cell myogenesis and tissue formation

In view of the clumps produced in the medium, the speed and resting time were adjusted. The results showed that when the speed decreased and the resting time increased, the number of clumps first increased and then decreased, and their volume continuously increased. Therefore, cell culture in a specific environment is hypothesized to promote myogenesis. Rotation speed and resting time were further adjusted to promote the generation of clumps, and muscle fiber markers were employed for staining identification.

**Fig. 4:**
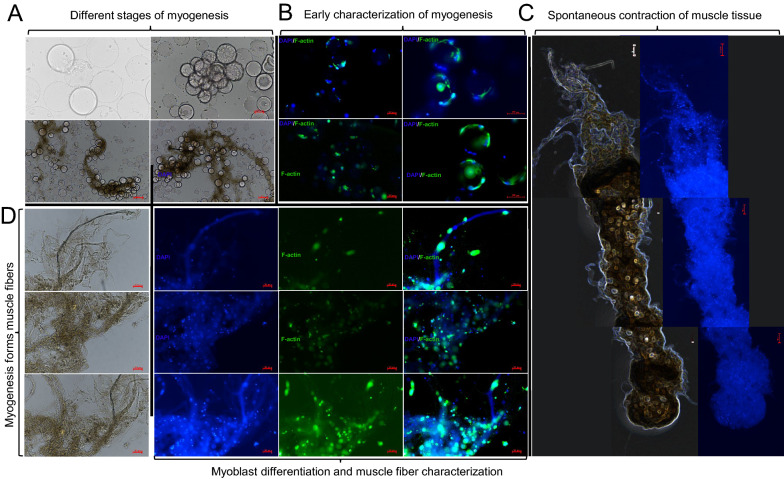
Long resting time promotes cell myogenesis. **A** Diagram of myoblasts at different myogenesis stages. **B** Early myogenesis identification results. **C** Myogenesis forms muscle fibers and then a tissue-like structure that contracts spontaneously. **D** Myogenesis promotes the differentiation of myoblasts and the formation of tissue-like structures

Identification through DAPI and F-actin revealed that these clumps were aggregates of filamentous muscle fibers. After further adjustment on rotation speed and resting time (Fig. 4), the cells first attached to the microcarriers to proliferate at a relatively high speed during myogenesis (Fig. 4A). When the proliferation reached a specific level, the cells gradually entered the contact inhibition stage. When the rotating speed of the spinner flask was reduced, the cells highly interacted during the contact process and then formed small clumps. With the increase in resting time, clumps gathered and generated large tissue mass, which in turn formed long muscle fibers and finally large-scale muscle tissues. The cultivated structured muscle tissues can contract spontaneously (Additional file 1: Video S1) and exhibits the normal contraction function of muscle tissues (Video is played at 5 × speed). For myogenesis verification, this study conducted the staining and identification of cells and muscle fibers at different culture stages (Fig. 4B) and assessed the morphology and organization of muscle fibers (Fig. 4D). The results showed that in the early tissue formation stage, the cells proliferated normally, DAPI staining was evident, cell viability was high, and F-actin staining brightness was low. When the rotation speed decreased, F-actin staining gradually deepened and increased in brightness. The amount of myogenesis also increased, and the single cells gradually formed myotubes and tissue structures with muscle fiber morphology. Therefore, tissues that have a muscle structure and contract spontaneously were finally synthesized (Fig. 4C).

### Microcarrier and cell ratio of 1:10 promotes large-scale cell culture

**Fig. 5:**
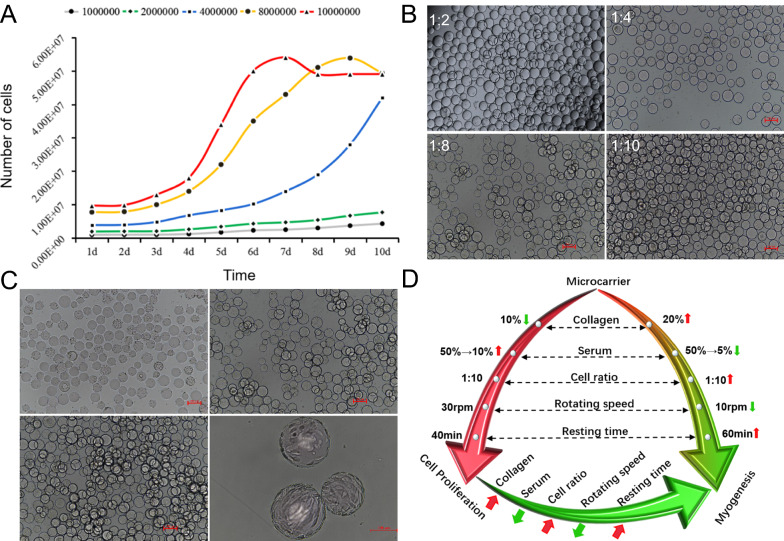
Number of inoculated cells and evaluation results of comprehensive optimization conditions. **A** Cell proliferation curve in spinner flask and microcarrier culture with different numbers of inoculated cells. **B** Morphology of inoculated cells attached to the microcarriers. **C** Comprehensive evaluation of the optimized conditions for the promotion of cell proliferation culture system. **D** Schematic of the adjustment of key parameters in different optimization schemes (large-scale cell proliferation system and myogenic system)

Cell proliferation has three stages: incubation, exponential, and plateau. Cells show an S-shaped growth trend during microcarrier-based culture. To explore the optimal seeding quantity for microcarrier-based cell culture, this work configured a specific concentration of microcarriers, calculated the number of microcarriers, and mixed different proportions of microcarriers and cells to explore the number of cells suitable for microcarrier-based culture. The results showed (Fig. 5A) significant differences in the number of inoculated cells with different ratios during proliferation. The cells with inoculation ratios of 1:1 and 1:2 failed to enter the exponential phase after 10 days of culture. In accordance with its proliferation trend, it will take at least 20 days to enter the exponential phase, which is not conducive to the large-scale proliferation of cells. For the 1:2 inoculation ratio, the average number of adherent cells per microcarrier was less than 1 (Fig. 5B). When the number of cell inoculation increased and the microcarrier and cell ratio was 1:4, the cells proliferated normally and entered the exponential phase to achieve effective proliferation. At this time, the cell proliferation curve had not entered the plateau phase on the 10th day. Although this ratio can achieve large-scale cell expansion, the process requires a long time. By contrast, cell proliferation for 1:8 and 1:10 ratios was significantly better than that for the other groups. These two groups can enter the exponential growth phase on the 4th day. However, due to the difference in the number of initial seeding cells, the 1:8 ratio group reached the plateau on the 9th day and the 1:10 ratio group on the 7th day. The cell inoculation system with 8- to tenfold microcarrier content is suitable for large-scale cell cultures using microcarriers and spinner flasks. In terms of cell attachment and proliferation status (Fig. 5B), is no significant difference was observed between the two culture systems. From an industrial perspective, the tenfold microcarrier amount is highly suitable. Given that the cycle from cell seeding to growth inhibition is 7 days, the industrialization process is greatly shortened. Research on cell inoculation systems promotes large-scale cell expansion and provides a theoretical basis and industrial guidance for cell proliferation in the production of cell-based meat. After the above optimization conditions were combined and all optimization schemes were adjusted, the cell adhesion efficiency was significantly increased, and the cell proliferation efficiency was significantly improved (Fig. 5C). This work successfully optimized the conditions of microcarrier pretreatment, serum conditions, spinner speed, resting time, medium addition, and number of inoculated cells (Fig. 5D) and explored the optimal conditions for myoblast culture using microcarriers and spinner flasks. The result revealed that pretreatment of microcarriers plays an important role in cell proliferation. The CG co-incubated microcarriers show better biocompatibility and cell adhesion than those treated with CG alone. The highest cell proliferation efficiency can be obtained when the number of inoculated cells is a quarter of the maximum carrying capacity of the microcarriers. At the beginning of the culture, the co-incubation of serum, cells, and microcarriers can improve the efficiency of cell adhesion and cell viability. A resting time of 40 min is beneficial to the rapid attachment and proliferation of cells, and a rotation speed of 30 rpm can maintain the suspended state of the microcarriers without its shearing force affecting cell proliferation. Increasing the CG concentration to more than 20%, reducing the final serum concentration to less than 10%, increasing the number of inoculated cells, reducing the rotating speed to less than 10 rpm, and extending the resting time to more than 60 min can effectively promote the myogenesis of myoblasts and promote changes from cells to tissues. Through the single-factor variable method, this study established an optimized process that is suitable for cell cultures using microcarriers and spinner flasks. The formed muscle fibers with normal muscle tissue functions were not synthesized through any medical or biological background modification or gene-level editing but simply through the physical methods of spinner flasks and microcarriers. This research provides a new direction for the treatment of muscle atrophy-related diseases in medical research and a new method for cell-based meat production.

### Discussion

The traditional method for microcarrier-based cell culture is to mix cells and microcarriers. The cells are cultured simply and continuously by adjusting the rotation speed of the spinner flask (Jin et al. 2014; Wang et al. 2018). Increasing the rotation speed of the spinner flask could increase the contact probability of cells and microcarriers; however, this process also increases shear forces, reduces cell viability (He et al. 2019), and is detrimental to cell attachment to microcarriers. In addition, cell proliferation efficiency is inhibited at an indeterminate speed. In cell culture, cell proliferation is affected by conditions such as cell type, cell viability, medium type, serum concentration, cell culture microenvironment, and culture methods. Therefore, a large-scale culture system for myoblasts is urgently needed.

In cell culture, a high serum concentration can effectively ensure the viability and proliferation ability of cells (Curtis and Forrester, 1984; Hoshiba et al. 2018), but the cost will increase accordingly. Improving cell viability (Petridou et al. 2007) and proliferation efficiency is the prerequisite to ensure large-scale cell culture without increasing the costs. The serum concentration of normal cell culture is 10%. On the one hand, serum concentration during the initial culture improves the biocompatibility of the microcarriers. On the other hand, it increases the viability of cells and promotes the efficiency of cell attachment. In this work, when most of the cells had attached to the microcarriers within 24 h, the culture medium was gradually increased to dilute the serum concentration to 10% to ensure low culture cost and increased cell attachment and proliferation efficiency.

In spinner flask cell culture (Sucosky et al. 2004), different rotation speeds greatly influence the shear force of the cells (Gupta et al. 2016; Shinde et al. 2021), which in turn affects their attachment and proliferation. In the optimized scheme, the initial speed of 5 rpm/min for the spinner flask was gradually increased at a rate of 5 rpm/min. The upper, middle, and lower three layers of the spinner culture medium were aspirated, and the number of microcarriers was equally distributed to these layers. At this time, the rotation speed was the minimum to suspend the microcarriers. The rotation speed can be increased without intensifying the shear force and affecting cell proliferation on the microcarriers (Jeske et al. 2021) to achieve the weightless suspension of the microcarriers, reduce the speed as much as possible (LIOVIC et al. 2012), and ensure the normal proliferation of cells while avoiding shedding.

In conventional spinner flask cell culture, the spinner flask program is launched to suspend the microcarriers upon the addition of cells and medium. At this time, the principle of cell attachment is to increase the contact probability of cells and microcarriers during the rotation of the culture medium and promote the attachment of cells to the surface of the microcarriers for proliferation. However, the efficiency of cell attachment is low. In the optimized solution, the microcarriers and cells were rotated for thorough mixing. When rotation was stopped, the cells fell and attached to the surface of the microcarriers under gravity. After resting time, rotation was restarted to mix the unattached cells and microcarriers and then abruptly discontinued. After many rotation/resting cycles, the cells can attach and proliferate efficiently (Ponnuru et al. 2014). The resting time during rotation/stationary has an important effect on cell attachment and proliferation. A short time promotes cell proliferation, and a long time enhances myogenesis. In addition, the resting time must be adjusted according to cell viability and attachment status. Depending on different needs, this parameter can promote the efficient attachment of cells, provide prerequisites for cell proliferation, and enhance myogenesis in cell differentiation.

Medium optimization was carried out through successive addition. The optimization principle is based on cell number and medium consumption degree. Cell proliferation is divided into incubation, exponential, and plateau periods (Hahn et al. 1968; Schmelzle and Hall, 2000). The incubation period must be relatively long during spinner flask culture, and the medium in the early stage must be added many times and in small amounts to improve the attachment and viability of the cells. In the exponential phase, the amount of added medium can be increased to either meet the nutrients required by the cells or dilute the cytotoxins metabolized by the cells (Stokes et al. 1999; Wagner and Rollinghoff, 1978). When being added to the complete system, one-third of the medium must be replaced according to the cell proliferation status for 1–2 days to ensure large-scale proliferation.

The number of inoculated cells (Gregorio et al. 2016; Masuda and Wakisaka, 1973) is the key to determining the efficiency of cell culture using spinner flasks and microcarriers. When the number of inoculated cells is extremely small, the efficiency of cell proliferation is low. While the number of inoculated cells is extremely large, the efficiency of microcarrier utilization is low, resulting in increased cost (Singh et al. 2010). For optimization, the maximum number of cells that a microcarrier can support for growth was first determined. Afterward, the average number of cells that can be seeded on a microcarrier and the total number of cells seeded into the culture system were calculated. This process ensures cell proliferation efficiency and microcarrier utilization efficiency. Finally, the influence of the number of inoculated cells on cell proliferation and culture time was examined, and the number of inoculated cells during spinner flask culture was inferred.

## Supplementary Information

**Additional file 1.** Video of muscle fiber formation and spontaneous contraction (5x playback speed).

## Data Availability

Data sharing is not applicable to this article as no datasets were generated or analysed during the current study.
